# Glycated Albumin Triggers an Inflammatory Response in the Human Airway Epithelium and Causes an Increase in Ciliary Beat Frequency

**DOI:** 10.3389/fphys.2021.653177

**Published:** 2021-04-23

**Authors:** Moira L. Aitken, Ranjani Somayaji, Thomas R. Hinds, Maricela Pier, Karla Droguett, Mariana Rios, Shawn J. Skerrett, Manuel Villalon

**Affiliations:** ^1^Department of Medicine, School of Medicine, University of Washington, Seattle, WA, United States; ^2^Department of Pharmacy, School of Medicine, University of Washington, Seattle, WA, United States; ^3^Department of Physiology, Faculty of Biological Sciences, Pontificia Universidad Católica de Chile, Santiago, Chile

**Keywords:** airways, airway disease, glycated albumin, inflammation, cytokine, human, ciliary beat frequency

## Abstract

The role of inflammation in airway epithelial cells and its regulation are important in several respiratory diseases. When disease is present, the barrier between the pulmonary circulation and the airway epithelium is damaged, allowing serum proteins to enter the airways. We identified that human glycated albumin (GA) is a molecule in human serum that triggers an inflammatory response in human airway epithelial cultures. We observed that single-donor human serum induced IL-8 secretion from primary human airway epithelial cells and from a cystic fibrosis airway cell line (CF1-16) in a dose-dependent manner. IL-8 secretion from airway epithelial cells was time dependent and rapidly increased in the first 4 h of incubation. Stimulation with GA promoted epithelial cells to secrete IL-8, and this increase was blocked by the anti-GA antibody. The IL-8 secretion induced by serum GA was 10–50-fold more potent than TNF_α_ or LPS stimulation. GA also has a functional effect on airway epithelial cells *in vitro*, increasing ciliary beat frequency. Our results demonstrate that the serum molecule GA is pro-inflammatory and triggers host defense responses including increases in IL-8 secretion and ciliary beat frequency in the human airway epithelium. Although the binding site of GA has not yet been described, it is possible that GA could bind to the receptor for advanced glycated end products (RAGE), known to be expressed in the airway epithelium; however, further experiments are needed to identify the mechanism involved. We highlight a possible role for GA in airway inflammation.

## Introduction

Airway epithelial cells participate in host defense by generating a wide variety of cytokines and chemokines that initiate or amplify acute and chronic inflammation by mediating the recruitment, activation, and survival of inflammatory cells within the airway (Standiford et al., 1990; Marini et al., 1992; Levine, 1995). Interleukin 8 (IL-8) is a pro-inflammatory molecule that is found in high concentration in the bronchial alveolar lavage (BAL) of patients with airway inflammatory diseases like cystic fibrosis (CF) (Konstan et al., 1994; O’Sullivan and Fredman, 2009), asthma (Lamblin et al., 1998), and chronic obstructive pulmonary disease (COPD) (Keatings et al., 1996; Hollander et al., 2007) and in the nasal mucosa of patients with allergic rhinitis (Cui et al., 2015). Respiratory epithelial cells (or cell lines) produce IL-8 in response to stimulation with IL-1α or TNFα or with other stimuli including neutrophil elastase, viruses, bacteria, and bacterial products (Standiford et al., 1990; Nakamura et al., 1992; Kwon et al., 1994; Levine, 1995). IL-8 is thought to play a major regulatory role in the airways as a potent chemo-attractant of polymorphonuclear granulocytes (PMNs) (Nakamura et al., 1991).

In airway diseases such as CF, asthma, and respiratory infections including viral infections notably SARS-CoV-2, the epithelial barrier is injured causing increased permeability and markedly greater plasma and serum protein movement across this barrier (McElvaney et al., 1992), a possible mechanism leading to a worse outcome in COVID-19 patients. Macromolecules of very different size and charge (i.e., 60-kDa albumin and the 700-kDa α_2_-macroglobulin) have been demonstrated to move equally across all barriers that exist between the venular compartment and the mucosal surface of airways (Svenson et al., 1995). The airway epithelium also participates in host defense through mucociliary clearance (MCC) which is designed to remove bacteria and contaminant particles from entering the lung parenchyma (Knowles and Boucher, 2002). MCC is determined by the frequency of ciliated cells and the rheological properties of the mucus released by secretory cells (van der Baan, 2000). The efficiency of MCC is affected by airway inflammation, which in turn induces the release of local pro-inflammatory molecules resulting in mucus hypersecretion or ciliary dysfunction (Seybold et al., 1990; Cowley et al., 1997; Waugh and Wilson, 2008; Schmid et al., 2010; Koblizek et al., 2011).

Limited understanding exists regarding the precise roles of serum molecules in the inflammatory response of the airway epithelium affecting MCC. We conducted the study herein to identify the protein in serum leading to this pro-inflammatory effect. We hypothesized that this protein induces increased airway inflammation and will cause functional effects such as on ciliary function, which plays an important role in chronic airway diseases.

## Materials and Methods

### Human Airway Epithelial Cells

We obtained nasal mucosa samples of 22 non-CF patients (mean age 47 years, range: 19–71 years); bronchial mucosa sample from one CF patient (33 years, cystic fibrosis genotype F508del homozygous); and adenoid tissue from non-CF patients (mean age 7 years, range: 3–12 years) who had undergone surgery for adenoid hypertrophy.

We also used an immortalized cell line from CF nasal polyp airway epithelial cells (CF 1–16 cells) with a homozygous F508del genotype, a gift from Dr. Christine Halbert, Fred Hutchinson Cancer Research Center, Seattle, WA, United States (Halbert et al., 1996). Tissue acquisition was approved by the ethic committee of the Pontificia Universidad Católica de Chile and the Human Subjects Review Committee of the University of Washington.

#### Nasal and Bronchial Mucosa Samples

Nasal epithelial cells were isolated from the mucosa by methodologies previously described (Standiford et al., 1990; Nakamura et al., 1991; McElvaney et al., 1992; Levine, 1995). Primary cultures from adenoid explant epithelial cells were prepared in Rose chambers as previously described (Gonzalez et al., 2013).

Human epithelial cells were isolated from the mucosa by methodologies previously described (Nakamura et al., 1991; McElvaney et al., 1992; Aitken et al., 1993, 1995; Levine, 1995; Gras et al., 2010; Gonzalez et al., 2013). Briefly, cells were rinsed and plated onto Vitrogen (Collagen Biomedical, Palo Alto, CA, United States)-coated 12-well plates (Costar, Cambridge, MA, United States) and cultured in 3.0 mL keratinocyte serum-free medium (KSFM) (Gibco BRL, Grand Island, NY, United States) with 5% fetal calf serum (FCS) (HyClone, Logan, UT, United States). After 12–24 h, the medium was changed to KSFM with 5 ng/mL epidermal growth factor (Gibco BRL, Grand Island, NY, United States) and 50 μg/mL bovine pituitary extract (Gibco BRL) until confluent (4–14 days). Experiments were performed on confluent cells. The monolayers were rinsed three times with either Hank’s balanced salt solution (HBSS) (H9269, Sigma, St Louis, MO, United States) or KSFM and incubated with KSFM under various experimental conditions.

#### Adenoid Tissue

Primary cultures were prepared to obtain explants of epithelial cells in Rose chambers as previously described (Gonzalez et al., 2013). Briefly, small pieces of adenoid tissue (2–4 mm) were washed with HBSS and rinsed in NHS media (137 mM NaCl, 5.09 mM KCl, 1.14 mM Na_2_HPO_4_, × 2 H_2_O, 0.18 mM KH_2_PO_4_, 0.923 mM MgCl_2_ × 6H_2_O, 0.91 mM CaCl_2_ × 2H_2_O, 4.07 mM NaHCO_3_, 21.5 mM glucose, pH 7.4) supplemented with 1% vitamins, 1% essential amino acids, 1% non-essential amino acids, and 1% pyruvate and antibiotics (neomycin 0.2 mg/mL and penicillin 0.12 mg/mL) (all these reagents: Invitrogen Corp, NY, United States). Small pieces of epithelium were placed on a cover glass pretreated with 0.1% gelatin (G9391, VWR Scientific, Radnor, PA, United States) and then placed in Rose chambers. Explants were covered with a sterile dialysis membrane pretreated with the NHS culture medium. The Rose chambers were filled with 2 mL of NHS medium, which contain 10% of horse inactivated serum (Biological Industries, Israel) (pH 7.2–7.4), and the explants were incubated at 37°C. The culture media within the Rose chambers were renewed every 48 h. After approximately 7 days, patches of epithelial cells with synchronized activity were obtained.

### Stimulation of Human Airway Epithelial Cells

Nasal human epithelial cell cultures were incubated in KSFM supplemented with commercial pooled serum (AB-Human Serum from Life Technologies, Biocompare, South San Francisco, CA, United States), single-donor serum, or plasma-derived serum, at concentrations ranging from 0.1 to 50%. In the stimulation experiments, after specified time intervals, supernatants were harvested and stored at –70°C. In some experiments, cells were incubated with single-donor serum in the presence of a neutralizing human monoclonal antibody against GA (A717, Exocell, Inc., Philadelphia, PA, United States. RRID:AB_2225805) at a concentration of 10 pg/mL.

The different serums were prepared as follows: single-donor serum was prepared from blood from a healthy subject. Blood was allowed to clot at room temperature, spun at 1,000 × g for 15 min, and subsequently the serum fraction was stored at −70°C. For plasma-derived serum, whole blood (50 mL) from a single donor was collected into a chilled syringe (4°C) containing 5 mL of 3.8% sodium citrate. The citrated blood was then centrifuged at 30,000 × g for 20 min at 4°C. The plasma was removed and mixed with 1.0 M CaCl_2_ (1:50) and then incubated at 37°C for 2 h and centrifuged at 25,000 × g for 20 min at room temperature. Serum was collected and stored at −70°C.

### IL-8 ELISA in the Monolayer Cells

Measurement of IL-8 was performed using “sandwich” enzyme-linked immunosorbent assay (ELISA). A 96-well plate was coated overnight with 100 μL of monoclonal antibody against human IL-8 (I2519, Sigma, RRID:AB_260157) at a concentration of 0.5 μg/mL, rinsed with PBS-Tween 20, and blocked with 2% BSA. The samples were then diluted, run in triplicate, and allowed to incubate at room temperature for 2 h. The plates were washed three times with PBS-Tween 20. A polyclonal antibody conjugated to horseradish peroxidase (HRP) was added to the plate, incubated, and washed. The chromogenic substrate 3,3′,5,5′-tetramethylbenzidine was added, and the absorbance was read at 450 nm. Unknowns were compared to serial dilution standards (0–2,000 pg/mL). IL-8 measurements are expressed in ng/mL considering 1 × 10^6^ cells per culture well.

### IL-8 ELISA in Rose Chamber Adenoid Cells

IL-8 production was measured from supernatants which were stored at −20°C until used. We used ELISA KIT Quantikine^®^ (minimum detectable dose: 3.5 pg/mL, D8000C, R&D Systems, Minneapolis, MN, United States). IL-8 measurements are expressed in ng/mL/mg of total protein with approximately 3 × 10^6^ cells per culture/mL.

### Identification of the Serum Factor (Glycated Albumin) That Increased IL-8 Secretion

Chromatography of Human Serum on DEAE Cellulose, pH 7: Ion exchange chromatography of human serum was performed on a 12-mL DEAE cellulose column (Whatman Specialty Products, Fairfield, NJ, United States) equilibrated in 50 mM NaCl, 10 mM phosphate, pH 7.5. Three milliliter of human serum dialyzed in equilibration buffer was added to the column, washed with seven column volumes of buffer, and collected in four equal fractions (zero absorbance in fourth fraction). A linear 50-mL salt gradient of 50–1,000 mM NaCl (in 10 mM phosphate, pH 7.5) was used to elute proteins from the column, and 0.6-mL fractions were collected. The salt concentration was monitored with an osmometer. The protein concentration was measured at 280 nm with a Beckman DU/64 spectrophotometer with a micro cuvette. Between 85 and 90 percent of the IL-8 secretory activity of primary non-CF nasal epithelial cells was found in the pre-gradient wash buffer fractions; these fractions were pooled and concentrated.

### Protein G Column

Immunoglobulins were removed from the pooled active fractions with a protein G Sepharose column (P3296, Sigma). A 5.0-mL column was washed in buffer containing 137 mM NaCl, 10 mM phosphate, pH 6.5. Serum or DEAE flow-through was dialyzed against the column buffer and applied to the column. The flow-through was collected along with 10 column volumes of wash buffer. Bound IgG was eluted with 100 mM of glycine, pH 2.5, and immediately neutralized with 0.5 N NaOH to pH 6.5. The flow-through and the eluted immunoglobulins were concentrated to their original volume with Millipore Ultrafree-15 centrifugal filters and dialyzed against 137 mM NaCl, 10 mM phosphate buffer, pH 7.5 (PBS). The majority of the IL-8 secretory activity of primary non-CF nasal epithelial cells was found in the flow-through and wash buffer fraction. No IL-8 secretion was found associated with the eluted immunoglobulin.

### Gel Filtration

The molecular size of the active protein from the previous steps was determined with a 12-mL S-200 Sephacryl gel column (GE17-0584-10, Sigma). The column was run with PBS. Two hundred microliters of concentrated protein was layered onto the gel, and 150-μL fractions were collected. The protein concentration was measured at 280 nm with a Beckman DU/64 spectrophotometer with a micro cuvette.

### Sequencing of IL-8-Stimulating Protein

**FIGURE 1:**
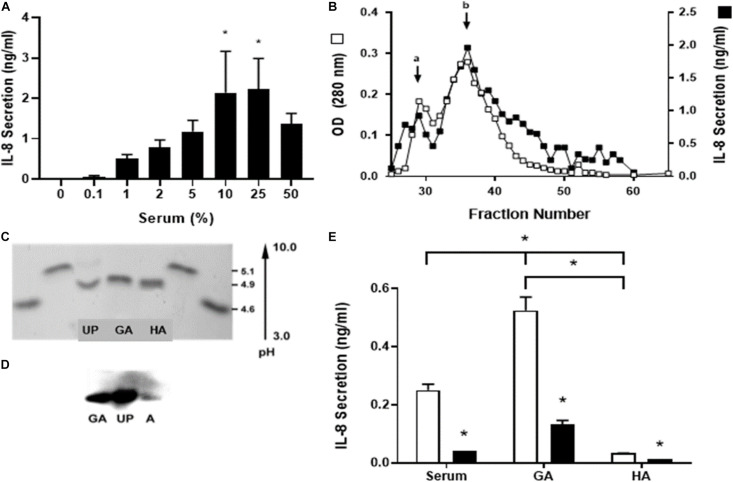
Identification of the serum component responsible for stimulation of IL-8 secretion. Human single-dose serum induced IL-8 secretion in a dose-dependent manner. **(A)** Nasal epithelial cells were incubated for 5 h with a different percentage of human single-dose serum, and IL-8 secretion was measured. Serum induced a dose-dependent IL-8 secretion with a maximum at 25%. * Indicates a statistically significant increase (*p* < 0.05) in IL-8 compared to control. **(B)** Different serum fractions obtained from an S-200 Sephacryl gel column were assayed for IL-8 secretion, and the optical densities at 280 nm were compared. Two peaks were obtained (a and b); the major peak (b) seems to be a dimmer of the active protein. These peptides from a trypsin digest of the peak protein were analyzed by MS and used in a BLAST search of human proteins to identify the unknown protein (UP). **(C)** UP was isoelectric focused with glycated albumin (GA) and human albumin (HA) to determine their isoelectric point (pI). Two standards used soybean trypsin inhibitor (4.6 pI) and bovine β-lactoglobulin A (5.1 pI) loaded at both sides from the bands of interest. UP has the same pI as GA and HA. **(D)** A Western blot with anti-glycated albumin antibody was carried out. GA, UP, and HA were recognized with the same molecular weight. **(E)** IL-8 secretion induced by serum and glycated albumin (GA) in CF1-16 cells was reduced with an anti-human GA antibody. The CF epithelial cell line was incubated for 5 h with commercial pooled human serum (serum), GA (2 mg/mL), or albumin (HA, 2 mg/mL) and an anti-human GA antibody (black bar). Open bars have no antibody. Serum and GA increased IL-8 secretion, which was blocked when cells were coincubated with the GA antibody. * Indicates a statistically significant (*p* < 0.05) reduction in IL-8 secretion in the presence of the antibody in all groups (open vs. black bar) or higher IL-8 secretion in cell cultures incubated with GA compared to serum or HA and serum compared to HA. *n* = 3 for experiments included in **(A,E)**. Experiments for **(B–D)** were repeated three times.

The identity of the unknown protein (UP) was determined by sequencing. The most active protein fraction in stimulating IL-8 secretion (fraction 38, Figure 1B) was electrophoresed on a native 7.5% acrylamide gel. Following electrophoresis, the gel was equilibrated for 5 min in a blotting buffer consisting of 100 mL of 10 mM CAPS buffer and 10% methanol, pH 11.0. The gel and a polyvinylidene difluoride (PVDF) membrane (Millipore, Bedford, MA, United States) were sandwiched and blotted for 45 min at 4°C. The membrane was washed and stained with Coomassie Blue R-250 for 5 min. Then, the membrane was destained in 50% methanol until the bands were clearly visible. The blotted membrane was rinsed in water and stored wet in a saran wrap at –20°C. The protein band of interest was sequenced from a trypsin digest of protein excised out of the PVDF and chromatographed by HPLC on a C18 column. Three peptides were separated out and sequenced using mass spectroscopy. The sequences obtained were used in a BLAST (RRID:SCR_004870)^1^ search of human proteins as a means to identify the protein.

### IEF (Isoelectric Focusing) Gel Electrophoresis

To further compare the UP with commercial human albumin (A1887, Sigma) and commercial glycated human albumin (A8301, Sigma), the iso-electro point of the three proteins was determined (Figure 1C). Equal amounts (6.9 μg) of the three proteins were run along with standards on a precast Bio-Rad IEF gel pH 3–10 (Bio-Rad, Hercules, CA, United States). The IEF standards were soybean trypsin inhibitor, 4.6 pI (10109886001, Sigma), and bovine milk β-lactoglobulin A, 5.1 pI (L3908, Sigma). The protein bands of interest were flanked on both sides by the standards in the gel. The gel was run for 3 h at various voltages; 1 h at 100 V, 1 h at 250 V, and 30 min at 500 V. The gel was fixed in 10% trichloroacetic acid (TCA) for 10 min. Excess ampholytes were removed by an additional overnight 1% TCA soak. To detect the protein bands, the gel was washed in water three times and stained with GELCODE Blue Stain Reagent (Pierce, Rockville, IL, United States).

### Western Blot With the Anti-GA Antibody

Human albumin (HA, A3782, Sigma), GA, and the UP were electrophoresed on a 4–20% native gradient gel (Figure 1D). Each lane contained 20 μg of protein. After electrophoresis, the proteins were transferred onto polyvinylidene difluoride (PVDF) membranes (Millipore, Bedford, MA, United States) and blocked with 2% gelatin in PBS pH 7.4 overnight. The blot was probed with a human monoclonal antibody against GA (A717, Exocell, Inc., Philadelphia, PA, United States. RRID: AB 2225805) diluted 1:500. It was then incubated with a secondary polyclonal antibody rabbit anti-mouse conjugated to HRP diluted 1:2,000 and visualized with ECL substrate (Amersham Biosciences Corp, Piscataway, NY, United States).

### Stimulation of CF1-16 Cells With GA, TNFα, and LPS

The levels of production measured through ligand stimulation with GA, TNFα, and LPS on epithelial cells were determined. CF1-16 cells were cultured with increasing concentrations of TNFα (0.2–20 ng/mL, T6674, Sigma), LPS (1–1,000 ng/mL, L3137 Sigma), and GA (0.2 μg/mL–2.0 mg/mL, A8301, Sigma). After a 5 h incubation at 5% CO_2_, 37°C, the supernatants were harvested and stored at –70°C until analyzed for IL-8 secretion by ELISA. Samples were run in triplicate and compared to a standard curve in the same methods described above. To calculate the EC50 and the extrapolated value for Vmax (maximum response), we used a standard model for fitting the data (non-cooperative activity). GraphPad Prism (RRID:SCR_002798)^2^.

### Ciliary Beat Frequency (CBF) Measurements

CBF was measured by microphotodensitometry as previously described (Hermoso et al., 2001; Barrera et al., 2004). Light fluctuations produced by the ciliary beat were sensed by a photodiode (FDS015, Thorlabs Inc., Newton, NJ, United States) placed on a phase microscope (Nikon 300 Diaphot Inverted Microscope). Signal was processed and displayed by a spectral analyzer (SAI-51C Honeywell, Charlotte, North Caroline, United States). Human adenoid epithelial cells in Rose chambers with spontaneous ciliary activity were washed with HBSS. Cultures were left with 2 mL of HBSS as supernatants. CBF was tested in monolayers of human epithelial cells in Rose Chambers. CBF was measured from 2 to 3 cells in each culture, and a CBF baseline was established for 30 min prior to adding a substrate. CBF was then measured every 15 min for up to 4 h. From each adenoid sample, we obtained around five cultures, each one with four or five explants surrounded by a monolayer of ciliated cells. In this study, we used 34 cultures of ciliated cells, obtained from seven patients. Each time course on CBF effect or Il-8 measurements was performed in 4–5 cultures.

### Statistical Analysis

Data were expressed as means ± standard error of the mean (SEM). Correlations between groups were calculated using the Spearman’s rank test. We used Student *t*-tests for mean comparisons between experimental groups, and linear regression was conducted to compare trends over time between groups. Dose–response curves on Il-8 secretion for GA, TNFα, and LPS were analyzed by one-way ANOVA followed by Holm–Sidak’s multiple-comparison test, using a square-root transformation of the original data. Graphics were performed using GraphPad Prism (see text footnote 2, RRID:SCR_002798). CBF data were analyzed following arcsin transformation. A *p* < 0.05 was considered statistically significant.

## Results

### Identification of the Serum Component Responsible for Stimulation of IL-8 Secretion

Serum from four healthy donors and commercial pooled AB serum all stimulated IL-8 secretion from primary airway epithelial cells at a range of 0.4–1.3 ng/mL/10^5^ cells.

We did not use pooled samples; rather, the individual whose serum had caused consistently the higher IL-8 response (1.7 ng/mL/10^5^ cells). Human nasal epithelial cells were incubated for 6 h at concentrations from 0.1 to 50% of the volume, and the level of IL-8 secretion was measured. Serum induced a dose-dependent IL-8 secretion with a maximum of 25% (Figure 1A).

To identify the serum component responsible for stimulating IL-8 secretion, an ion exchange chromatography of human serum was performed to identify the unknown protein (UP) (Figure 1B). When fractionated, two distinct peaks were identified: a major peak with a molecular weight of about 65 kDa (non-glycated albumin) and a minor peak at approximately 130 kDa (glycated albumin). In subsequent purifications, we noticed that the minor peak quantity was variable. It was postulated that the IL-8 released was associated with both peaks and that the minor peak could be a dimer or aggregate of the active UP. The most active fraction (fraction 38) was sent for sequencing, and the resultant sequence was used in a BLAST search against human proteins as a mean to identify the UP. The BLAST result indicated with a high probability that the UP was human serum albumin which has a molecular weight of ∼69 kDa which was similar to the UP weight (Figure 1C).

UP was isoelectric focused with glycated albumin (GA) and human albumin (HA) to determine their isoelectric point (pI). Two standards, soybean trypsin inhibitor (4.6 pI) and bovine β-lactoglobulin A (5.1 pI), were loaded at both sides from the bands of interest. UP have the same pI as GA and HA (Figure 1D).

A Western blot with equal amounts of anti-glycated albumin antibody was carried out. The results demonstrated that the UP and GA had a strong signal while HA had a very small response as expected, as the monoclonal antibody used targeted GA and thus may not react with non-glycated albumin. Further, all three proteins had equivalent electrophoretic mobility on the 7.7% SDS PAGE gel with a molecular weight of about 67 kDa (Figure 1E).

IL-8 secretion induced by serum and glycated albumin (GA) in CF1-16 cells was reduced significantly with an anti-human GA antibody (*p* = 0.01). CF1-16 epithelial cells were incubated for 5 h with commercial pooled human serum, GA (2 mg/mL) or human albumin (HA, 2 mg/mL), and with an anti-human GA antibody. Open bars have no antibody; black bars have an anti-human GA antibody. Serum and GA increased IL-8 secretion, which was blocked when cells were incubated with a GA antibody. HA was unable to increase IL-8 secretion supporting the need for glycation of albumin for this to occur (*p* = 0.24).

### Human Upper and Lower Airway Epithelial Cells Had Varying Responses in Il-8 Production With Human Serum Stimulation

**FIGURE 2:**
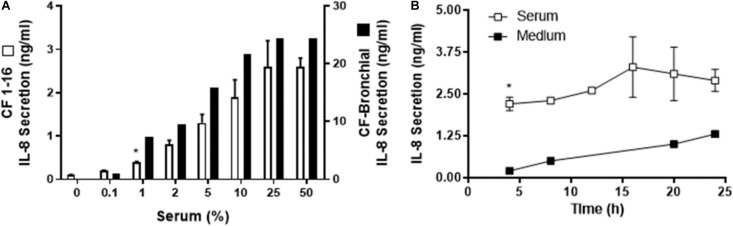
Human serum induced IL-8 secretion from a cystic fibrosis (CF) epithelial cell line. **(A)** Immortalized CF nasal epithelial cell line (CF 1–16) (open bars, *n* = 3) and CF bronchial epithelial cells (black bars, *n* = 2) were incubated for 6 h with increasing percentages of human serum, and IL-8 secretion was measured. Human serum induced a dose-dependent IL-8 secretion in both CF cell line and the CF bronchial cells. **(A)** * indicates the lowest % of serum capable of producing a significant increase in IL-8 production. CF bronchial epithelial cells’ response to serum to produce IL-8 secretion is almost one order of magnitude higher compared to the cystic fibrosis epithelial cell line. **(B)** CF 1–16 cells were incubated with 5% of single-donor serum (□, *n* = 3) or medium (■, *n* = 2), and IL-8 secretion (*n* = 3–4) was determined at 4, 8, 12, 16, 20, and 24 h. At 4 h of incubation, human serum induced a significant increase in IL-8 secretion compared to the control group. **(B)** * indicates a statistically significant (*p* < 0.05) increase in IL-8 secretion after 4 h of incubation with serum compared to the control group. No significant changes were observed for IL-8 secretion after longer period of incubation.

We wished to determine if the IL-8 secretion response was similar using the immortalized human CF cell line and the bronchial CF cells (Figure 2A). Cells were incubated for 6 h at concentrations from 0.1 to 50%, and the level of IL-8 secretion was measured. A dose response was demonstrated in all three cell types shown in human nasal cells (Figure 1A) and immortalized CF1-16 and CF bronchial cells (Figure 2A) with a maximal serum concentration of 25% (Figures 1A, 2A), and a maximum IL-8 secretion of 22.30 ng/mL in the CF bronchial cells.

Further, we wished to determine if immortalized CF 1-16 cells incubated with 5% of single-donor serum or medium over 4–24 h would show an IL-8 response (Figure 2B).

In the three epithelial cell types (human nasal epithelial cells, immortalized CF1-16 cell line, and CF bronchial epithelial cells), the ED_50_ for serum was 5, 5, and 3% with extrapolated maximums of 2.3, 2.8, and 24 ng/mL of IL-8, respectively. The magnitude of the IL-8 response in CF-bronchial cells was 10-fold higher compared to immortalized CF 1–16 airway epithelial cells (*p* = 0.02) (Figure 2A). A parallel experiment using plasma-derived serum free of platelet factor contamination demonstrated a similar IL-8 dose–response relationship (to a maximum of 10%) in airway epithelial cells (data not shown).

The time dependence of IL-8 secretion was determined using CF1-16 cell cultures (Figure 2B). CF1-16 cells were incubated with 5% of single-donor serum in KSFM, or with KSFM alone, and the supernatants were harvested at 4, 8, 12, 16, 20, and 24 h. By 4 h of incubation, human serum induced a significant increase of IL-8 secretion compared to the control group (Figure 2B). No significant changes were observed for IL-8 secretion after a longer incubation period in either group.

### IL-8 Secretion Induced by Glycated Albumin Compared to TNFα and LPS

**FIGURE 3:**
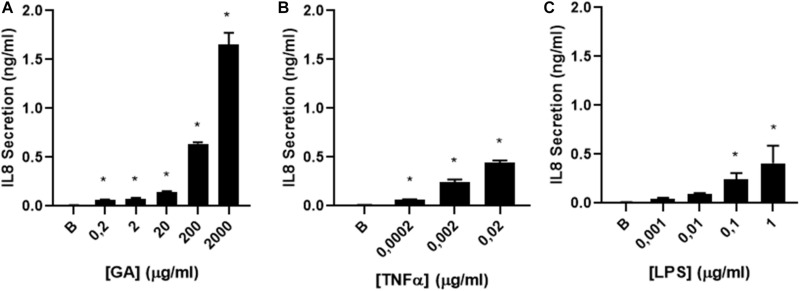
IL-8 secretion by glycated albumin (GA) compared to TNFα and LPS. **(A)** dose–response curve of IL-8 secretion was determined in CF1-16 cells induced by **(A)** GA (*n* = 3), **(B)** TNFα (*n* = 3), and **(C)** LPS (*n* = 3). All induced a dose-dependent IL-8 secretion, with a maximum effect on Il-8 production for TNF and LPS of 477.6 and 424 ng/mL, respectively, but GA was 4–5 times more effective, reaching a max effect of 2.0 ng/mL. The EC_50_ for LPS was 0.071 and 0.00193 μg/mL for TNF compared to 424 μg/mL for GA. * indicates a significant increase (*p* < 0.05) in IL-8 secretion compared to the background **(B)**.

The efficacy of GA to stimulate the airway epithelium to secrete IL-8 was comparatively assessed in CF 1–16 cells with other two pro-inflammatory molecules, TNFα and LPS. The concentrations of TNFα and LPS used were selected based on their pathological concentrations described in the literature (Demling et al., 2005; Kakazu et al., 2011). All induced a dose-dependent IL-8 secretion, with a maximum effect on Il-8 production for LPS and TNF of 0.419 and 0.471 ng/mL, respectively, but GA was over four times more effective, reaching a maximum effect of 2.0 ng/mL. The EC_50_ for LPS was 0.071 and 0.00193 μg/mL for TNF compared to 424 μg/mL for GA (Figure 3).

### Glycated Albumin Increased IL-8 Secretion and CBF in Human Adenoid Cell Cultures

**FIGURE 4:**
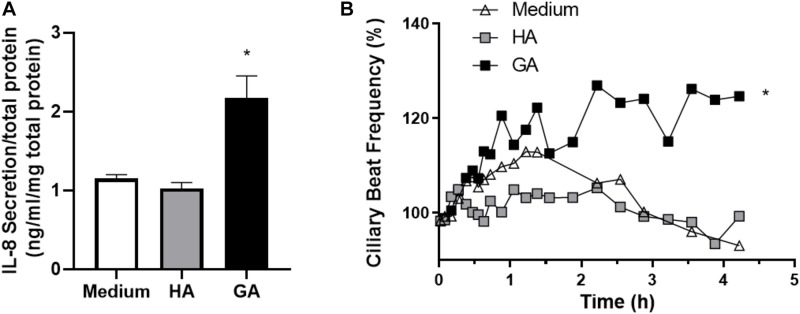
GA increased IL-8 secretion and CBF in human adenoid epithelial cell cultures. **(A)** Human primary adenoid epithelial ciliated cell cultures were incubated with culture medium, HA (2 mg/mL), or GA (2 mg/mL) for 3 h. GA increased the statistical significance of IL-8 secretion with respect to HA and medium. Values are expressed as ng/mL of IL-8 corrected by mg of protein in primary culture. *p < 0.05. **(B)** Time-course changes in CBF of non-CF human adenoid ciliated cell culture treated with GA (■, 2 mg/mL), HA (□, 2 mg/mL), and control solution (△, medium). The most significant effect upon CBF was observed after 2–4 h of incubation with GA. *n* = 4–5 cultures were used for each experimental point included in the Il-8 secretion experiments and the time course changes on CBF exposed to medium, HA, and GA.

Next, human primary adenoid epithelial ciliated cell cultures were used to measure IL-8 secretion following 3 h of incubation with culture medium (control), HA, and GA. An elevated level of IL-8 secretion only occurred in the cultures stimulated with GA, while there was no difference between control and HA treated cultures (*p* = 0.12) (Figure 4A). When ciliary beat frequency was assessed, a progressive increment was detected in response to GA incubation, compared to control or HA groups (*p* < 0.001 for GA group). Furthermore, the most significant effect upon CBF was observed following 2–4 h of incubation with GA (Figure 4B). CBF, expressed as area under the curve (AUC, background was subtracted), was significantly greater in the GA-treated cultures compared to HA and culture medium cultures (GA 139.40 ± 4.54; HA 66.58 ± 1.99; medium 79.67 ± 2.87, *p* < 0.05).

**FIGURE 5:**
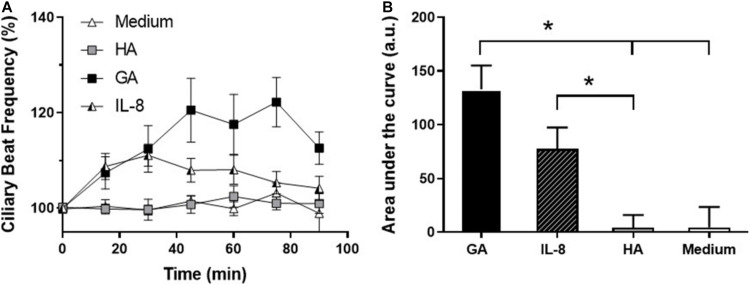
Effect of glycated albumin (GA) and IL-8 in CBF. **(A)** Time-course changes in CBF of human adenoid epithelial ciliated cells cultures treated with GA (■, 2 mg/mL), IL-8 (□, 10 nM), albumin (HA, ∘, 2 mg/mL), and culture medium (△). GA and IL-8 induced an increase in CBF in the first 30 min of exposure, but only the increase of CBF induced by GA was sustained. **(B)** Time course of CBF in **(A)** expressed as area under the curve (AUC). GA induced a statistically significant increase in CBF with respect to HA and medium, and IL-8 with respect to HA. *p < 0.05. *n* = 4–5 cultures were used for each experimental point included in the time-course changes on CBF exposed to medium, HA, Il-8, and GA.

The temporal relationship between the effect of GA and IL-8 secretion on CBF was examined by comparing the time changes in CBF induced by GA (2 mg/mL) and IL-8 (10 nM). GA and IL-8 both induced an increase in CBF in the first 30 min of exposure, but only the increase of CBF induced by GA remained elevated. In contrast, no changes were observed in CBF in cultures incubated with HA (2 mg/mL) or medium alone (Figures 5A,B).

## Discussion

We purified a human serum factor that increases IL-8 secretion from airway epithelial cells and, using molecular and mass spectrometric methods, identified it as human serum (glycated) albumin. Through comparative assessment, we demonstrated human serum glycated albumin (GA) to be a potent pro-inflammatory molecule with functional effects on the ciliary airway. GA was able to stimulate IL-8 secretion in different epithelial respiratory cell models, including primary cultures of human epithelial nasal cells, adenoid epithelial cells, CF bronchial cells, and an immortalized CF airway epithelial cell line. GA had a greater effect on IL-8 secretion compared with conventional pro-inflammatory molecules of TNF-α and LPS. Furthermore, both GA and IL-8 increased CBF in non-CF human adenoid epithelial cells.

GA is an advanced glycation end product (AGE) considered a pro-inflammatory molecule. AGEs are formed from condensation and oxidation processed between proteins and sugars (Sorci et al., 1833). Clinically, serum GA levels, used to detect early glycemic changes (Hoonhorst et al., 2016), may be twice the normal range in diabetes (Dolhofer and Wieland, 1980; Jones et al., 1983). IL-8 expression is significantly elevated in patients with type II diabetes (Herder et al., 2005). These higher levels of GA are recognized as a major cause of diabetes complications such as atherosclerosis, cardiovascular disorders, nephropathy, and chronic inflammation (Sorci et al., 1833). In addition, GA has been identified as important in the induction of inflammation in retinal epithelial cells and monoclonal antibodies against GA ameliorate diabetic nephropathy in a mouse model (Cohen et al., 1994). In endothelial cells, the presence of GA is deleterious, making cells more procoagulant and promoting inflammatory responses (Rubenstein et al., 2011). Using enzyme-linked immunoassay, GA-treated human retinal pigment epithelial cells secrete levels of IL-8 detectable within 4 h (Bian et al., 1996). In human retinal pigment epithelial cells and in vascular smooth muscle cells, GA induces the expression of IL-8 by a mechanism that involves activation of kinases, including PKC, and transcription factors such as NF-κβ (Chen et al., 2000; Bian et al., 2001; Dahrouj et al., 2015). NF-kβ induces the expression of inflammatory response-related mRNAs, such as TNFα (tumor necrosis factor α) and IL-6 (interleukin-6) (Svenson et al., 1995), inflammatory mediators that have been related with functional changes in cilia activity (Chen et al., 2000; Papathanasiou et al., 2008). Furthermore, kinases like PKC also modify the mechanism associated with cilia activity regulation by ATP (Barrera et al., 2004; Gonzalez et al., 2013).

In human primary airway epithelial ciliated cell cultures, we observed that GA induced an increase in IL-8 and CBF. Previous studies have shown that macromolecules, such as soluble hyaluronic acid, can increase CBF of tracheal ovine epithelial cells through an unknown membrane receptor (Lieb et al., 2000). Non-glycated HA was unable to modify CBF. Although the binding site of GA has not yet been described, it is possible that GA could bind to the receptor for advanced glycated end products (RAGE), known to be expressed in the airway epithelium (Milutinovic et al., 2012). RAGE regulates a number of cellular processes such as inflammation, regulation of cell mass, and cell mobility (Xie et al., 2013; Whitsett and Alenghat, 2014). Studies in retinal pigment epithelial cell cultures indicate that glycated albumin-induced breakdown of RPE function is mediated by RAGE and vascular endothelial growth factor (VEGF) receptor (del Campo et al., 2011). RAGE has been shown to be present in epithelial cells and contribute to allergic airway disease, as it can act as a mediator in one or more pro-inflammatory pathways that eventuate in the altered physiology seen in allergic airways disease (Milutinovic et al., 2012). RAGE knockout mice present a lower production of IL-8 and NF-kβ activation when they are exposed to pulmonary ischemia and reperfusion (Sternberg et al., 2008). In addition to binding AGEs, RAGE also is a signal transduction receptor for amyloid-β (Aβ) and S100/calgranulins, suggesting that RAGE could be involved in the recognition of a variety of macromolecules associated with the respiratory innate immunity response of the airway epithelium (Ma et al., 2007).

The CF 1-16 cell line in the study responded to human serum, increasing IL-8 secretion after 4 h of incubation. CF bronchial epithelial cells also responded to human serum, increasing IL-8 secretion, but CF bronchial cells appeared to be more responsive releasing 10 times more IL-8. Patients with CF suffer from chronic infections and severe inflammation and present high levels of pro-inflammatory molecules such as IL-8, IL-6, TNF_α_, and arachidonic acid metabolites (Nakamura et al., 1992; Khan et al., 1995; O’Sullivan and Fredman, 2009), indicating a possible pro-inflammatory state of these cells. In addition, CF patients with CF-related diabetes mellitus have a more rapid decline in lung function and an increased mortality rate (Milla et al., 2000). It is possible that increased GA in the airways of these patients enhances the inflammatory response in their lungs.

Previous studies have shown that TNFα induces IL-8 expression in cultured human airway epithelial cells (Kwon et al., 1994). Furthermore, LPS, a TLR4 agonist, increases IL-8 production through phosphorylation of p38 (Mizunoe et al., 2012). When we compared the effectiveness of GA to induce IL-8 secretion with TNFα and LPS, GA was 10 times more effective to induce IL-8 secretion, suggesting a critical role of GA to initiate an inflammatory response.

IL-8 induced an increase in CBF in adenoid epithelial cultures showing a correlation with the effect of GA in the first 30 min. The CXC chemokine receptor (CXCR-1/IL-8) has been found to be expressed in human small airway epithelial cells and to induce the release of IL-6 (Gras et al., 2010). Although the specific mechanism of Il-8 stimulation of CBF has not been identified, it has been reported that Il-8 can increase PLC and intracellular calcium levels, both well-known ciliary beat activators (Barrera et al., 2004). Murine primary sino-nasal cultures treated with the mouse homologue of IL-8 increased basal CBF at 24 and 48 h (Shen et al., 2012). However, IL-8 inhibits beta-agonist ciliary stimulation in bovine bronchial epithelial cells (Allen-Gipson et al., 2004). It is possible that GA and IL-8 affect CBF directly or, alternately, that IL-8 secretion induced by GA enhances CBF (Choi et al., 2010). Furthermore, it is possible that the initial activation of CBF induced by Il-8 could be reverted after longer periods of inflammation, with a deleterious effect upon mucociliary clearance.

Our results suggest that plasma exudation of serum molecules such as GA onto the airway epithelium could induce an inflammatory response, initially producing IL-8 secretion and modifying mucociliary transport by the effect on ciliary activity. A progression of this inflammatory response could involve the release of other chemotactic cytokines, an influx of polymorphonuclear neutrophils, and contribute to airway inflammation observed in chronic airway diseases (Reutershan and Ley, 2004). Future directions may include measurement of GA in several airway diseases, including SARS-CoV-2, and correlate GA levels with patient clinical outcomes. Recent studies have contributed to identifying the location of the receptor of advanced glycation end products (RAGE) in the airways, as a mediator of GA effects on the systemic inflammatory response (Hoonhorst et al., 2016). A study reported by Demling et al. (2005) reported that the RAGE receptor on alveolar epithelial type 1 cells (AT 1) was localized in the basolateral membrane, suggesting a morphological role for the receptor by strengthening the adherence of ATI cells to the alveolar basement membrane. In our study, we used different cellular epithelial cell types, including polarized and non-polarized cells, but we did not identify the specific location of the RAGE receptor. Future studies using double chambers to produce polarized cell cultures will be necessary to identify the RAGE receptor location and activation on the apical or basal membrane by GA inducing an inflammatory response, helping to identify potential blockers to prevent the deleterious effect of AGE, including GA, on the airway epithelium.

In our study, we used different epithelial cell types including polarized and non-polarized cells. However, we did not identify the specific location of the RAGE receptor. Future studies using double chambers to produced polarized cell culture will be necessary to identify the RAGE receptor location and activation on the apical or basal membrane using GA to induce an inflammatory response to assist in identifying potential blockers to prevent the deleterious effects of AGE including GA, on the airway epithelium.

In summary, our results suggest that plasma exudation of serum molecules such as GA onto the airway epithelium can induce an inflammatory response, initially producing IL-8 secretion and modifying mucociliary transport by the effect on ciliary activity. A progression of this inflammatory response could involve the release of other chemotactic cytokines, an influx of polymorphonuclear neutrophils, and contribute to airway inflammation observed in chronic airway diseases. Future directions may include measurement of GA in several airway diseases, including SARS-CoV-2, and correlate GA levels with patient clinical outcomes. Recent studies have contributed to our understanding by identifying the location of the receptor of advanced glycation end products (RAGE) in the airways, as a mediator of GA effects on the inflammatory response (Buckley and Ehrhardt, 2010; Hoonhorst et al., 2016). However, a study by Demling et al. (2005) reported that the RAGE receptor on the alveolar epithelial type I cells (AT I) was localized in the basolateral membrane, suggesting a morphological role for the receptor by strengthening the adherence of AT I cells to the alveolar basal membrane.

## Conclusion

In conclusion, our findings suggest that a serum protein, GA, may have an important role in the airway inflammatory response.

## Data Availability Statement

The raw data supporting the conclusions of this article will be made available by the authors, without undue reservation.

## Ethics Statement

The studies involving human participants were reviewed and approved by the University of Washington IRB. Written informed consent for participation was not required for this study in accordance with the national legislation and the institutional requirements.

## Author Contributions

MA, TH, SS, and MV planned the experimental designs. MA, RS, TH, MP, KD, MR, SS, and MV wrote the manuscript. MA and SS identified a serum factor that induced inflammation. TH and MP identified glycated albumin as the inflammatory protein. MP performed the dose escalation experiments. KD, MR, and MV performed the experiments on CBF. All authors contributed to the revision and final draft of the manuscript.

## Conflict of Interest

The authors declare that the research was conducted in the absence of any commercial or financial relationships that could be construed as a potential conflict of interest.

## Footnotes

1 http://blast.ncbi.nlm.nih.gov/Blast.cgi

2 http://www.graphpad.com/

## Abbreviations

BAL: Bronchial alveolar lavage
COPD: Chronic obstructive pulmonary disease
CBF: Ciliary beat frequency
CF: Cystic fibrosis
GA: Glycated albumin
HBSS: Hank’s balanced salt solution
HRP: Horseradish peroxidase
IL-8: Interleukin 8
IEF: Isoelectric focusing
KSFM: Keratinocyte serum-free medium
PMNs: Polymorphonuclear granulocytes
PVDF: Polyvinylidene difluoride
SEM: Standard error of the mean
TCA: Trichloroacetic acid
UP: Unknown protein.
